# Amphotericin B resistance correlates with increased fitness *in vitro* and *in vivo* in *Leishmania* (*Mundinia*) *martiniquensis*

**DOI:** 10.3389/fmicb.2023.1156061

**Published:** 2023-04-06

**Authors:** Chonlada Mano, Aphisek Kongkaew, Pongsri Tippawangkosol, Pradya Somboon, Sittiruk Roytrakul, Pascale Pescher, Gerald F. Späth, Chairat Uthaipibull, Adisak Tantiworawit, Padet Siriyasatien, Narissara Jariyapan

**Affiliations:** ^1^Department of Parasitology, Faculty of Medicine, Chiang Mai University, Chiang Mai, Thailand; ^2^Animal House Unit, Faculty of Medicine, Chiang Mai University, Chiang Mai, Thailand; ^3^National Center for Genetic Engineering and Biotechnology (BIOTEC), National Science and Technology Development Agency, Pathum Thani, Thailand; ^4^Institut Pasteur, INSERM U1201, Université Paris Cité, Unité de Parasitologie Moléculaire et Signalisation, Paris, France; ^5^Thailand Center of Excellence for Life Sciences (TCELS), Bangkok, Thailand; ^6^Department of Internal Medicine, Faculty of Medicine, Chiang Mai University, Chiang Mai, Thailand; ^7^Center of Excellence in Vector Biology and Vector-Borne Disease, Department of Parasitology, Faculty of Medicine, Chulalongkorn University, Bangkok, Thailand

**Keywords:** *Leishmania*, *Leishmania martiniquensis*, leishmaniasis, fitness, Amphotericin B, drug resistance, relapse, Thailand

## Abstract

Amphotericin B (AmpB) deoxycholate is the available first-line drug used to treat visceral leishmaniasis caused by *Leishmania* (*Mundinia*) *martiniquensis*, however, some cases of AmpB treatment failure have been reported in Thailand. Resistance to drugs is known to affect parasite fitness with a potential impact on parasite transmission but still little is known about the effect of resistance to drugs on *L. martiniquensis*. Here we aimed to gain insight into the fitness changes occurring after treatment failure or *in vitro*-induced resistance to AmpB. *L*. *martiniquensis* parasites isolated from a patient before (LSCM1) and after relapse (LSCM1-6) were compared for *in vitro* and *in vivo* fitness changes together with an *in vitro* induced AmpB-resistant parasite generated from LSCM1 parasites (AmpBRP2i). Results revealed increased metacyclogenesis of the AmpBPR2i and LSCM1-6 strains (AmpB-resistant strains) compared to the LSCM1 strain and increased fitness with respect to growth and infectivity. The LSCM1-6 and AmpBRP2i strains were present in mice for longer periods compared to the LSCM1 strain, but no clinical signs of the disease were observed. These results suggest that the AmpB-resistant parasites could be more efficiently transmitted to humans and maintained in asymptomatic hosts longer than the susceptible strain. The asymptomatic hosts therefore may represent “reservoirs” for the resistant parasites enhancing transmission. The results in this study advocate an urgent need to search and monitor for AmpB-resistant *L. martiniquensis* in patients with relapsing leishmaniasis and in asymptomatic patients, especially, in HIV/*Leishmania* coinfected patients.

## Introduction

*Leishmania* parasites cause a group of anthropozoonoses called leishmaniases, which are important neglected tropical diseases. Metacyclic promastigotes, the infectious form of the parasite, are inoculated to mammalian hosts during the blood meal of infected insect vectors, where they are efficiently phagocytosed by cells of the mononuclear phagocytic system, notably macrophages. There, promastigotes differentiate into amastigote forms whose intracellular replication will ultimately cause the immunopathologies associated with the different forms of leishmaniasis, which include cutaneous, mucocutaneous, and visceral leishmaniases (VL). Their clinical spectrum ranges from asymptomatic infection or self-limiting cutaneous lesions to lethal disseminated infections depending on the species of *Leishmania* parasite and the host’s immune response. *L. martiniquensis* is a newly emerging causative agent of human leishmaniasis first reported from Martinique Island (French West Indies) (Desbois et al., 2014) and later found in many countries including Thailand. Most human cases present clinical features of VL, however, HIV-coinfected patients also develop disseminated cutaneous leishmaniasis (Pothirat et al., 2014; Chiewchanvit et al., 2015). In the absence of vaccines against human leishmaniasis, chemotherapy is still the only alternative to tackle the disease despite the emergence of drug resistance (World Health Organization, 2022).

Amphotericin B deoxycholate is a widely used antifungal and antiprotozoal compound. It binds to ergosterol molecules presenting in the membrane of fungi or trypanosomatids, which causes depolarization of the membrane and alters membrane permeability towards cations, water, and glucose molecules resulting in ions leakage and ultimately leading to cell death. This drug has been introduced for the treatment of VL in antimonial unresponsive patients from Bihar in India, but two clinical AmpB-resistant *Leishmania donovani* strains have already been reported (Srivastava et al., 2011; Purkait et al., 2012). Most leishmaniasis cases in Thailand are treated with AmpB (Jariyapan et al., 2018) but recurrence of the disease after treatment has been reported in some cases for both immunocompetent and immunocompromised patients, including HIV-infected individuals (Osatakul et al., 2014; Siriyasatien et al., 2016).

Resistance phenotype to overcome drug treatment often comes with fitness changes, fitness being defined as a complex integrated skill that allows microorganisms to successfully replicate in a defined environment (Natera et al., 2007). *Leishmania* parasites are highly adaptive microorganisms as they must survive and replicate within different environments depending on their hosts (insect and mammal) to be transmitted. In this context, the ability of *Leishmania* parasites to resist the activity of an antileishmanial drug is shown to have an impact on their fitness in one or both hosts which can vary depending on the drug and the parasite species. Differences in parasites viability, growth, metacyclic promastigote generation, and infectivity in laboratory animals have been reported for different species or strains of *Leishmania* parasites resistant to different anti-leishmanial drugs (García-Hernández et al., 2015; Hendrickx et al., 2015; Turner et al., 2015). Experimentally generated *L. donovani* strains that are resistant to single anti-leishmanial drugs (AmpB, miltefosine (MIL), paromomycin (PMM), and trivalent antimony (Sb^III^)) or to drug combinations (AmB-MIL, AmB-PMM, AmB-Sb^III^, MIL-PMM, and Sb^III^-PMM) present a higher promastigote survival rate in conditions of starvation, a higher tolerance to heat shock and pH stress, and an increased survival rate for *in vitro* macrophage infections compared to their corresponding wild type (García-Hernández et al., 2015). MIL-resistant *L. major* populations generated *in vitro* using stepwise selection exhibited a similar growth rate and response to stress as the wild-type parasites but, despite the enhancement of metacyclogenesis these parasites show virulence attenuation *in vitro* and *in vivo* infection assays and decrease survival rates in the natural sandfly vector (Turner et al., 2015). In contrast, comparative phenotypic analysis of a matched pair of an *L. donovani* PMM-susceptible, WT parent strain, and its derived PMM-resistant strain revealed no impact of the PMM-resistance phenotype on parasite fitness regarding promastigote growth, metacyclogenesis, and *in vitro* and *in vivo* infectivity (Hendrickx et al., 2015).

So far, no data on the fitness of drug-resistant *L. martiniquensis*, a member of the new subgenus *Mundinia*, are available. We have reported three cases of leishmaniasis caused by *L. martiniquensis* in northern Thailand (Pothirat et al., 2014; Chiewchanvit et al., 2015) that showed relapse of the disease after the first treatment with AmpB. We successfully isolated the parasites from bone marrow samples and/or skin biopsy samples collected from a patient before treatment (LSCM1) and after relapse (LSCM1-6) and could generate an *in vitro*-induced AmpB-resistant strain (AmpBRP2i). Their respective fitness phenotypes were analyzed by comparing (i) *in vitro* promastigote growth, (ii) differentiation into infectious metacyclic forms in culture (metacyclogenesis), (iii) *in vitro* infectivity and multiplication in mouse peritoneal exudate macrophages (PEMs), and (iv) *in vivo* infectivity in BALB/c mice. We uncovered *in vitro* and *in vivo* increased fitness that correlated with the AmpB resistance of *L. martiniquensis*. This information reveals an important, latent public health threat that calls for an in-depth epidemiological survey and molecular analysis of the underlying drug resistance and fitness mechanisms.

## Materials and methods

### Ethics statement

The study was approved by the ethics committee of the Faculty of Medicine, Chulalongkorn University (COA No. 467/2021), and approval to use mice was obtained from the Ethics Committee on Animal Use of the Laboratory Animal Center, Chiang Mai University, Chiang Mai, Thailand (COA No. 2562/MC-0009).

### Parasite strain and culture

*Leishmania martiniquensis* parasites, LSCM1 (MHOM/TH/2012/LSCM1, wild-type), and LSCM1-6 (MHOM/TH/2017/LSCM1-6, relapse) strains were used in this study. *Leishmania martiniquensis* LSCM1 was obtained from a VL patient with no known underlying immunodeficiency. After being treated with AmpB (1 mg/kg/day) for 21 days at the first admission, the patient was in remission (Pothirat et al., 2014). However, relapse occurred about 1 year after the first treatment. Over 5 years, the patient had been given at least six courses of the treatment with AmpB. In 2017, 5 years after the first relapse we successfully isolated and cultured *L*. *martiniquensis* LSCM1-6 parasites from bone marrow aspirate.

To avoid loss of parasite virulence, the parasite strains, LSCM1 and LSCM1-6, were inoculated in BALB/c mice and recovered from the liver or spleen of the infected mice 4 weeks post-infection. Isolated parasites were then cultured for two to three passages in sterile Schneider’s Insect medium (SIM) (Sigma-Aldrich, St Louis, MO, United States), pH 6.8 supplemented with 10% (v/v) heat-inactivated fetal bovine serum (hiFBS) (Life Technologies-Gibco, Grand Island, NY, United States). Cultured parasites (approximately 1 × 10^7^ cells/mL) were cryopreserved in 7.5% (v/v) glycerol in the culture medium and stored in liquid nitrogen. Cryopreserved promastigotes were used for this study, and the maximum passage number used was seven after cryopreservation. For routine cultivation, parasites were maintained in the SIM complete medium supplemented with 25 μg/mL gentamycin sulfate (Sigma-Aldrich, St Louis, MO, United States) at 26°C. Promastigotes were sub-passaged to a fresh medium every 4 days to maintain the growth and viability of the parasites.

### Drug

AmpB was purchased from Gibco (Life Technologies-Gibco, Grand Island, NY, United States) as a 250 μg/mL solution solubilized in sodium deoxycholate. The stock solution of AmpB was stored at −20°C and used within 12 months.

### Animal

Eight to twelve-week-old inbred male BALB/c mice (*Mus musculus*), purchased from Nomura Siam International Co., Ltd., Bangkok, Thailand, weighing approximately 20 g were used. Mice were housed in the animal facilities at the Laboratory Animal Center, Chiang Mai University under standard conditions of temperature (25 ± 2°C), 12 h light/dark cycle, and fed with a standard pellet diet and water *ad libitum.*

### Isolation of mouse peritoneal exudate macrophages

The isolation of PEMs was performed as described by Zhang et al. (2008) with some modifications. Eight to twelve-week-old female BALB/c mice were injected *via* an intraperitoneal route with 1 mL of 3% (w/v) Brewer thioglycolate (Himedia, India) solution in PBS. PEMs were harvested after 48 h by peritoneal lavage with 2% hiFBS-RPMI1640 ice-cold medium (GE Healthcare Life Science-HyClone, South Logan, UT, United States) containing 1% penicillin–streptomycin (PenStrep; Sigma, United Kingdom). PEMs were collected by centrifugation (×500 g, 4°C, 10 min) and then resuspended in RPMI medium containing 10% (v/v) hiFBS. The viability of PEMs was estimated using trypan blue staining solution (Sigma-Aldrich, St Louis, MO, United States) in an improved Neubauer chamber (Precicolor, HBG, Germany) under light microscopy.

### *In vitro* drug susceptibility on promastigotes

Promastigote viability in the presence of the drug was evaluated using alamarBlue^®^ assay (Thermo Fisher Scientific, MA, United States) and performed in flat-bottomed 96-well tissue-culture plates. Each well was filled with 50 μL of the logarithmic phase culture of promastigotes (2 × 10^6^ cells/mL) and incubated at 26°C for 1 h before adding AmpB. Parasites were exposed to 50 μL of AmpB over a range of concentrations in two-fold drug dilutions (0.0016–25.6 μg/mL). After 48 h of incubation, 10 μL of alamarBlue^®^ reagent was added to each well and continuously incubated for 24 h. The concentration of resorufin in the parasite-drug mixture was measured using a spectrophotometer at a wavelength of 570 and 600 nm. The optical density in the absence of drugs was set as 100% control. Drug susceptibility was determined by calculating the half-maximal inhibitory concentration IC_50_ values from the nonlinear concentration-response curves using GraphPad Prism version 9.1 software (Graphpad Software Inc., San Diego, CA, United States) and the results were expressed as the mean ± standard deviation (SD) of three independent experiments.

### Resistance selection on promastigotes

A stepwise process previously described by Al-Mohammed et al. (2005) and García-Hernández et al. (2012) was used to select an AmpB-resistant line from *L. martiniquensis* promastigotes (LSCM1) with some modifications. Briefly, the selection was initiated with promastigotes (2 × 10^6^ cells/mL) starting from 0.025 μg/mL of AmpB corresponding to half of the IC_50_ value determined for the LSCM1 promastigote (0.05 μg/mL) to 1.0 μg/mL. After each selection step, log-phase (day 3) promastigotes (2 × 10^6^ cells/mL) were sub-passaged in the SIM medium supplemented with 20% (v/v) hiFBS and increasing concentrations of AmpB in stepwise increments. This experiment was carried out in flat-bottomed 24-well plastic tissue-culture plates (ThermoFisher Scientific, Jiangsu, China) with a final volume of 1 ml and the plates were maintained at 26°C. At each concentration of AmpB, the promastigotes were maintained until a growth rate was similar to the LSCM1 control culture. This process was applied until reaching the maximum concentration of the drug allowing parasite growth. Then, three single clones of the AmpB-resistant promastigotes, namely, AmpB-Resistant Promastigote clone 1 (AmpBRP1), AmpB-Resistant Promastigote clone 2 (AmpBRP2), and AmpB-Resistant Promastigote clone 3 (AmpBRP3), were selected from the *in vitro* derived AmpB-resistant line by limiting dilution to 1 cell/mL. The three resistant clones were sub-passaged in the SIM, pH 6.8 supplemented with 10% (v/v) hiFBS without AmpB for 20, 30, and 40 passages after the selection. For the 20th, 30th, and 40th passages the corresponding resistance indexes (IC_50_ of each clone divided by IC_50_ of the LSCM1) were calculated. The AmpBRP2 resistant clone that showed high stability of IC_50_ value and resistance index at passage 40 was selected. To avoid the impact of the long-term culture of the selected clone (40 passages) on infectivity, the parasites with stationary phase promastigotes (at 120 h) were used to infect BALB/c mice for 4 weeks. As described above parasites were isolated from the liver or spleen of the infected mice and cultured and the IC_50_ value and resistance index of the isolated parasites were determined to check for stability of AmpB resistance before cryopreservation for further use.

### *In vitro* promastigote growth

The growth profile of the LSCM1, AmpBRP, and LSCM1-6 parasites was assessed by a direct counting method using an improved Neubauer chamber. To generate growth curves, stationary phase promastigotes were inoculated at exactly 1 × 10^6^ cells/mL in 5 mL of the SIM complete medium and incubated at 26°C. Parasite density was determined every 24 h for 10 consecutive days (24 h to 240 h). The average promastigote density at each time point was calculated and used to draw the final growth curves using GraphPad Prism version 9.1 software. The doubling time was calculated during the exponential growth of the parasites, i.e., between 48 and 72 h of culture. All experiments were carried out in duplicate from three independent experiments. Results were expressed as mean ± SD.

### Morphological assessment for metacyclogenesis

Promastigote morphology was evaluated microscopically to assess metacyclogenesis. The cell body size (length and width) and flagellum lengths were measured. Promastigotes were considered metacyclic when the body length was ≤12.5 μm, body width ≤ 1.5 μm, and flagellum length > body length (Chanmol et al., 2019). Promastigotes of the LSCM1, AmpBRP, and LSCM1-6 were cultured in the SIM, at 26°C. From 72 h to 240 h of the cultivation, 10 μl of each promastigote suspension was stained with 10% (v/v) Giemsa solution. Images were acquired under Olympus CX41RF light microscope (Tokyo, Japan) at ×1,000 magnification and the cell body and flagellum lengths of ≥200 parasites were measured using DP2-SAL Firmware Ver.3.3.1.198, software. The percentage of metacyclic form was calculated at each time point of the culture. Results were expressed as mean ± SD and were based on three independent experiments in duplicates.

### *In vitro* infection and evaluation of intracellular amastigote multiplication

PEMs harvested from BALB/c mice were tested for cell viability using trypan blue. PEMs with cell viability above 95% were used. A total of 2.5 × 10^5^ cells of PEMs in 500 μL RPMI medium with 10% (v/v) hiFBS were plated in round coverslips placed in 24-well tissue culture plates and incubated at 37°C and 5% CO_2_ for 24 h. Nonadherent cells were washed out with a pre-warmed RPMI medium. To estimate the number of parasites for a ratio of 1:10 adherent cells was counted after the 24 h incubation. Then, the adherent cells were infected with the stationary phase promastigotes (at 120 h) of the LSCM1, AmpBRP, or the LSCM1-6 at the ratio of 1:10. Live/dead staining with trypan blue was used to correct for the variable number of dead promastigotes in the different cultures. Parasites with cell viability above 99% were used in this experiment. After 3 h of incubation, extracellular promastigotes were then removed by washing twice with a pre-warmed RPMI medium. Coverslips were fixed with absolute methanol for 10 s, Giemsa’s-stained for 30 min, and visualized under the Olympus CX41RF light microscope (×1,000 magnification). To evaluate the level of infection, at least 200 macrophages were counted in 10 randomly selected microscopic fields in duplicate. The percentage of infected macrophages (infection rate) and the average number of intracellular parasites per macrophage were determined. In addition, the infection index was calculated by multiplying the percentage of infected macrophages by the average number of intracellular parasites per macrophage to account for the overall intracellular parasite burden.

Evaluation of amastigote multiplication was performed every 24 h from 24 h to 120 h post-infection using the same process. To allow comparison between the different strains, correction for the baseline infectivity was made based on the infection ratio at 24 h post-infection (T_0_). The amastigote multiplication ratio was calculated from the average number of intracellular amastigotes at T_x_ (the evaluated time after 24 h post-infection) divided by the average number of intracellular amastigotes at T_0_ (Chanmol et al., 2019). Results were expressed as mean ± SD and based on three independent infection experiments, each performed in duplicate.

### *In vivo* infectivity

For each parasite strain, 42 BALB/c mice were used. Animals were intraperitoneally injected with 2 × 10^7^ stationary phase promastigotes (at 120 h) resuspended in 200 μL of PBS. In the control group, six mice were injected with 200 μL of PBS. The infected mice were monitored weekly for cachexia, fatigue, ascites, scabs or skin lesions, hepatomegaly, and splenomegaly and their body weight was recorded using a balance (Sartorius TE313S Talent Analytical Balance, Sartorius AG, Goettingen, Germany). At 1-, 3-, 7-, 28-, 84-, and 168-days post infection (dpi), six animals from each group were sacrificed. In each animal, the liver, spleen, and bone marrow were collected separately under sterile conditions. The liver and spleen samples were weighed. Parasite burden in the liver, spleen, and bone marrow was determined by the impression smear method and limiting dilution assay.

For the impression smear method, parasite burden in each organ expressed as Leishman Donovan units (LDU) was calculated using the formula according to Stauber et al. (1958). The LDU values correspond to the number of amastigotes per 1,000 nucleated cells multiplied by the organ weight (g).

For limiting dilution assay, parasite burden was quantified in these tissues as previously described by Intakhan et al. (2020). The parasite load was calculated from the mean of reciprocal positive titers divided by the weight of the homogenized cross-section and calculated as the number of parasites per gram of organ. Genomic DNA from all samples was also extracted for the detection of *L. martiniquensis* DNA by PCR using 70IRD/70IRM primers for the 3 ´ untranslated region (3 ´-UTR) of the heat shock protein 70 (type I) gene (*HSP70-I*) (Jariyapan et al., 2021).

### Statistical analysis

All statistical analyses were performed using GraphPad Prism version 9.1 software. The statistical differences among the LSCM1, *in vitro* derived AmpB-resistant, and LSCM1-6 strains and between the different time points within one group were evaluated using two-way ANOVA, followed by Bonferroni post-hoc comparison tests. The IC_50_ values were established from the dose–response curves using the GraphPad Prism software. All experiments were performed in triplicate and considered statistically significant if the *p* value was <0.05.

## Results

### *In vitro* generation of AmpB-resistant clones

**Figure 1:**
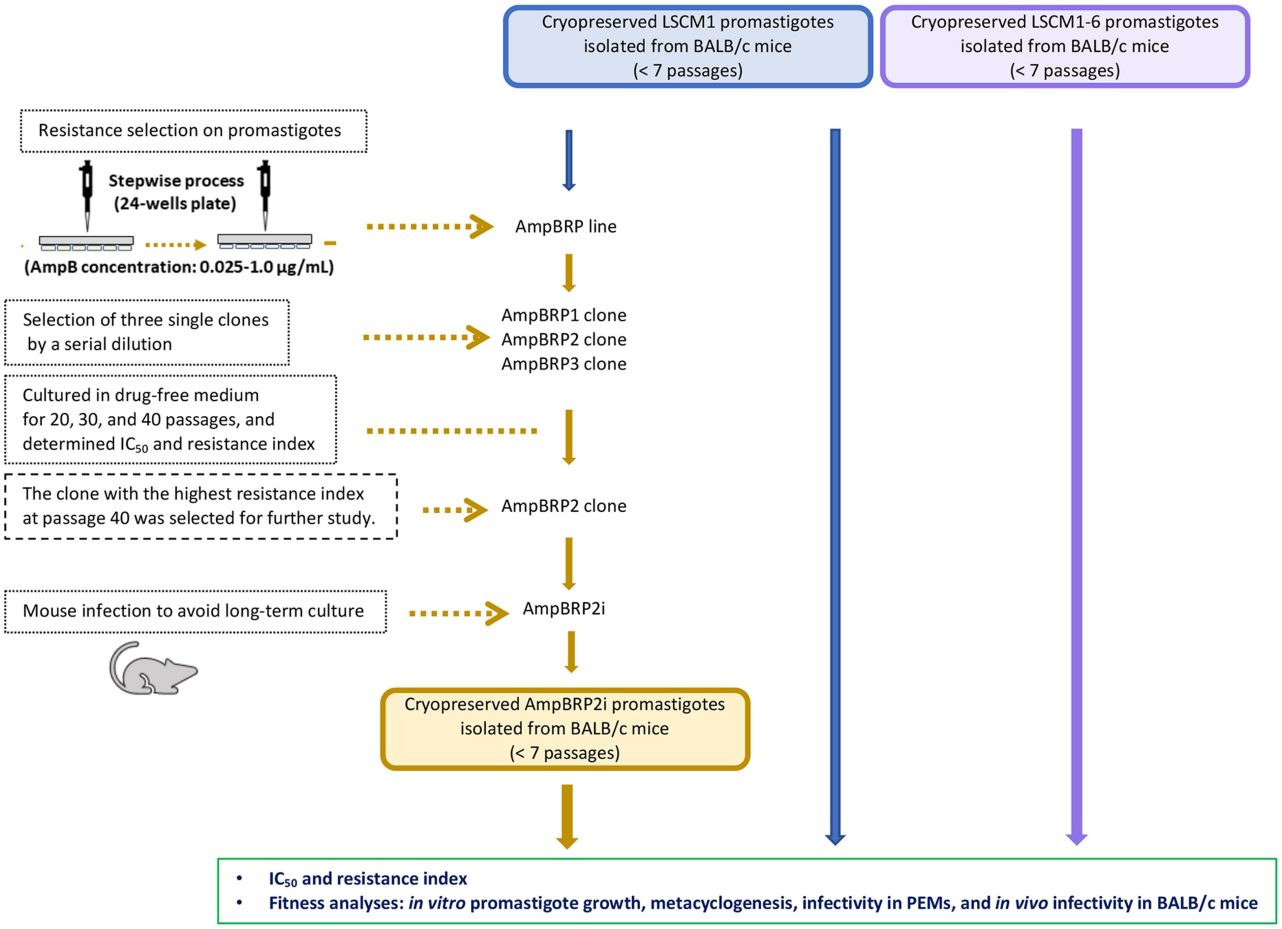
Schematic diagram of resistance selection for an AmpB-resistant clone on promastigotes and an overview of the comparative fitness of the LSCM1, AmpB-resistant clone, and LSCM1-6.

To select AmpB-resistant lines of *L. martiniquensis*, promastigotes of the LSCM1 strain were exposed to AmpB at a starting concentration of 0.025 μg/mL, which was stepwise increased to induce drug resistance (Figure 1). The final concentration of AmpB that did not affect the normal growth of the *in vitro* derived AmpB-resistant promastigotes was 1.0 μg/mL. Overall, the selection time lasted 260 days. Three single clones, AmpBRP1, AmpBRP2, and AmpBRP3, were selected from the *in vitro* derived AmpB-resistant line by limiting dilution to 1 cell/mL. In every ten *in vitro* passages the drug susceptibility of the selected AmpB-resistant clones was tested to assess the stability of AmpB resistance. The cutoff value for AmpB-resistant strains was set at 0.55 μg/ml. At passage 20, the IC_50_ and resistance index of the AmpBRP clones were ranging from 1.23 to 1.59 μg/mL and from 22.4 to 28.9, respectively (Table 1). The IC_50_ and the resistance index for the three selected clones slightly decreased (statistically insignificant) after being maintained in the drug-free medium for more than 20 passages. Both the IC_50_ and resistance index of these AmpBRP clones at passages 20, 30, and 40 were higher than that of the LSCM1 input population (Table 1). Based on the highest resistance index observed at passage 40 (26.3), the AmpBRP2 clone was selected for further study.

**Table 1:** Promastigote susceptibility to AmpB for *L. martiniquensis* LSCM1, and *in vitro* derived AmpBRP clones after removal from drug pressure. Results are expressed as mean ± SD based on three independent replicates.

Parasite strain	Passages 20		Passages 30		Passages 40	
	IC_50_ (μg/mL)	Resistance index*^a^*	IC_50_ (μg/mL)	Resistance index	IC_50_ (μg/mL)	Resistance index
LSCM1	0.055 ± 0.02	1	0.054 ± 0.01	1	0.052 ± 0.01	1
AmpBRP1	1.23 ± 0.22	22.4	1.17 ± 0.07	21.7	1.11 ± 0.08	21.3
AmpBRP2	1.59 ± 0.01	28.9	1.49 ± 0.15	27.6	1.37 ± 0.29	26.3
AmpBRP3	1.55 ± 0.01	28.2	1.52 ± 0.03	28.1	1.25 ± 0.17	24.0

a Resistance index = IC_50_ of each AmpBRP clone ÷ IC_50_ of LSCM1.

To avoid the impact of the long-term culture on the infectivity of the AmpBRP2 clone, the parasites at passage 40 were used to infect BALB/c mice for 4 weeks. The stability of resistance to AmpB of the AmpBRP2 parasites recovered from the spleen of one infected mouse (further called AmpBRP2i) was evaluated. The IC_50_ value and resistance index of the isolated AmpBRP2i were 0.191 ± 0.02 μg/mL and 3.7, respectively (Table 2), inferior to the values of the input parasites but still in the same range as the relapsed strain, LSCM1-6. The AmpBRP2i strain, therefore, was used for the *in vitro* and *in vivo* phenotypic comparisons with the susceptible wild-type parent strain, LSCM1, and the relapse strain, LSCM1-6 (Figure 1).

**Table 2:** Promastigote susceptibility to AmpB for the LSCM1, AmpBRP2i, and LSCM1-6 strains before and after mouse infection. Results are expressed as mean ± SD based on three independent replicates.

Parasite strain	Before mouse infection		After the 1st mouse infection		After the 2nd mouse infection (day 28)	
	IC_50_ (μg/mL)	Resistance index	IC_50_ (μg/mL)	Resistance index	IC_50_ (μg/mL)	Resistance index
LSCM1	0.052 ± 0.01	1	0.052 ± 0.01	1	0.053 ± 0.01	1
AmpBRP2i	1.370 ± 0.29	26.3	0.191 ± 0.02	3.7	0.190 ± 0.02	3.6
LSCM1-6	0.147 ± 0.03	2.8	NA^a^	NA	0.154 ± 0.08	2.9

a NA = not available.

### *In vitro* promastigote growth

**Figure 2:**
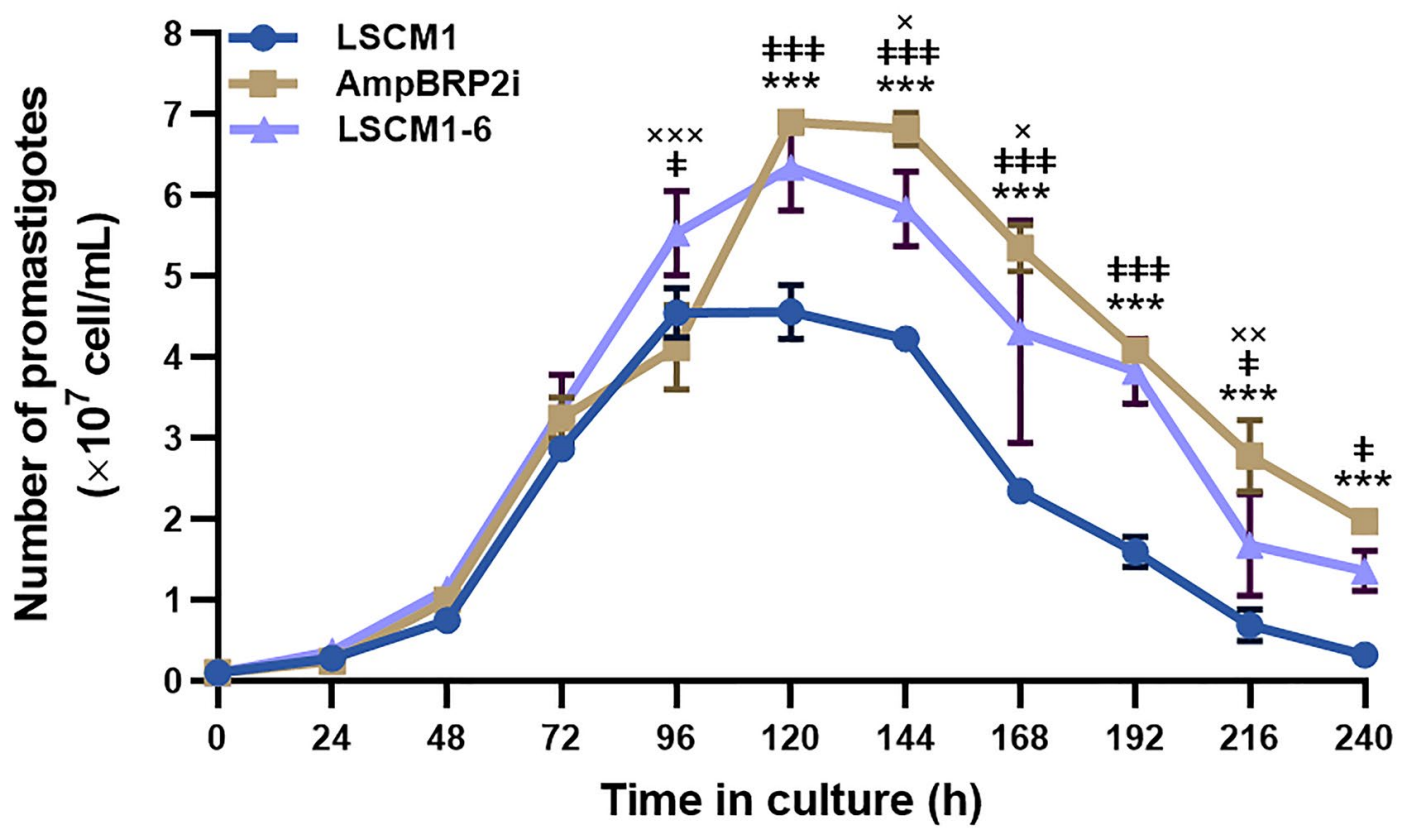
Growth curves of the *L. martiniquensis* LSCM1, AmpBRP2i, and LSCM1-6 promastigotes cultured in the SIM supplemented with 10% (v/v) hiFBS and 25 μg/ml gentamycin sulfate, pH 6.8 at 26°C. All parasites were inoculated at 1×10^6^ parasites from stationary growth phase/mL and were counted daily until day 10 (240 h). Statistically significant differences between LSCM1 and AmpBRP2i are indicated as *** = *p* < 0.001; LSCM1 and LSCM1-6 are indicated as **ǂǂǂ** = *p* < 0.001, **ǂǂ =** *p* < 0.01, and **ǂ =** *p* < 0.05; AmpBPR2i and LSCM1-6 are indicated as **××× =** *p* < 0.001 and **×× =** *p* < 0.01, and **× =** *p* < 0.05.

LSCM1, AmpBRP2i, and LSCM1-6 strains were compared for their growth as promastigotes in the SIM complete medium at 26°C (Figure 2; Supplementary Table S1). Promastigotes of the LSCM1 reached their peak at 96 h (4.56 × 10^7^ cells/mL), and then started to decline to approximately 0.32 × 10^7^ cells/mL at 240 h. AmpBRP2i promastigotes reached their peak and entered the stationary phase at 120 h with a density of 6.91 × 10^7^ cells/mL, and then at 144 h the parasite concentration started to decline to 1.97 × 10^7^ cells/mL at 240 h. LSCM1-6 promastigotes reached a plateau at 96 h (5.54 × 10^7^ cells/mL) and sustained the stationary phase until 144 h (5.84 × 10^7^ cells/mL) before the continuous decrease until the end of the experiment. The average promastigote densities of the AmpBRP2i and the LSCM1-6 were significantly greater than that of the LSCM1 from 120 h to 240 h or from 96 h to 240 h, respectively. However, the average promastigote density of the LSCM1-6 was significantly lower than that of the AmpBRP2i at 144, 168, and 216 h. In summary, the AmpB-resistant parasites reached a higher cell density than the parental strain LSCM1.

### Metacyclogenesis

**Figure 3:**
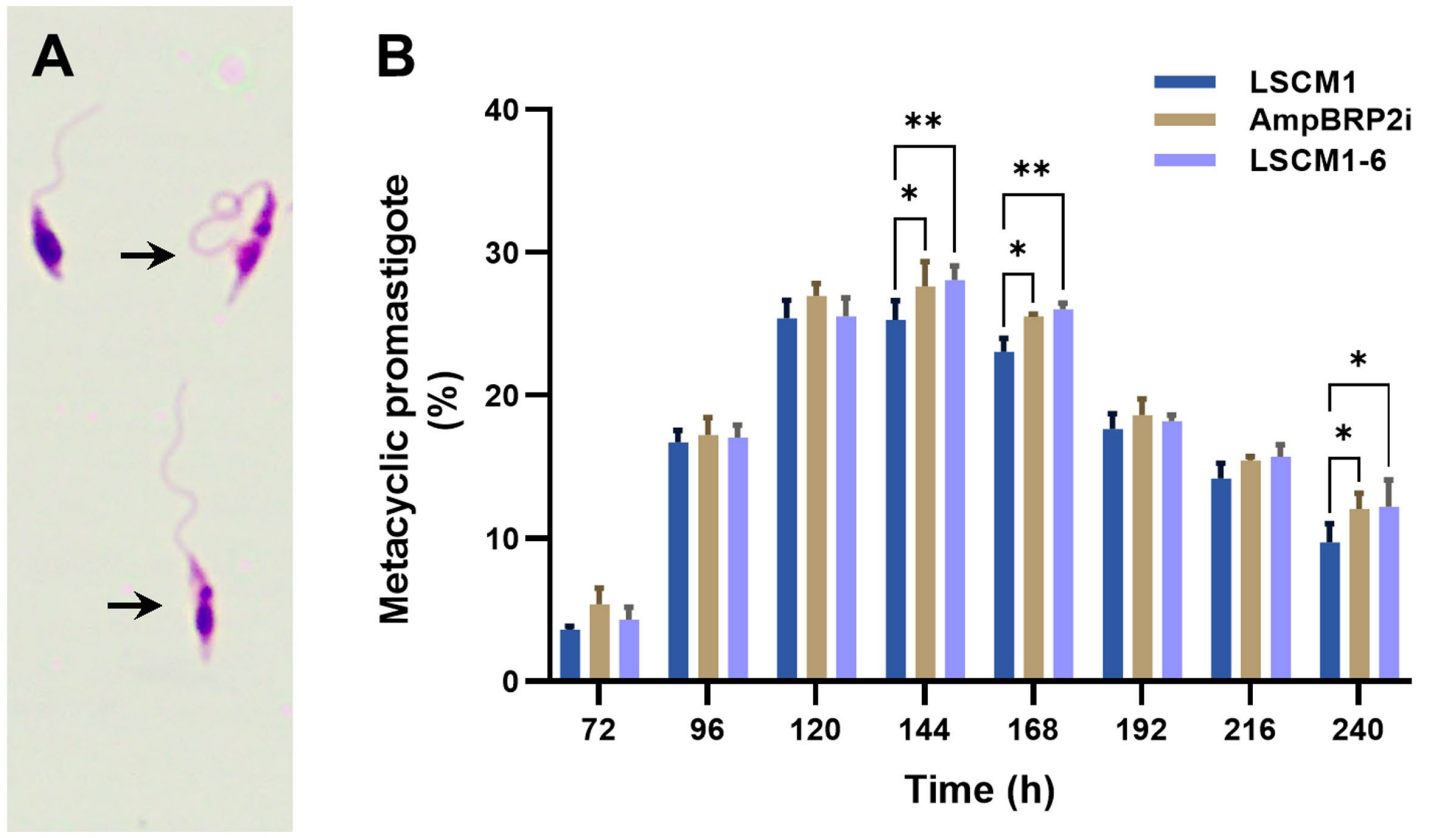
**(A)** Metacyclic promastigotes of *L. martiniquensis* (arrows). **(B)** Metacyclogenesis of the LSCM1, AmpBRP2i, and LSCM1-6 promastigotes. Morphometric analysis was conducted by microscopy using the criteria defined by Chanmol et al. (2019) from 72 to 240 h of culture. Statistically significant differences among the three groups are indicated as follows: ** = *p* < 0.01; and * = *p* < 0.05.

Metacyclic promastigotes of the LSCM1, AmpBRP2i, and LSCM1-6 strains were evaluated based on morphological criteria (Figure 3A). The percentage of metacyclic promastigotes of the three strains was similar at 72 h (3.6–5.4%), increased by approximately 6-fold at 120 h (25.4–27%) and sustained for almost 3 days (Figure 3B; Supplementary Table S2). The number of LSCM1 metacyclic-like parasites reached a maximum at 120 h with 25.4% and then gradually dropped to 9.7% after 240 h of culture. For both AmpBRP2i and LSCM1-6, the percentage of metacyclic promastigotes reached its peak at 144 h with 27.6 and 28.1% respectively, and then decreased gradually to 12% at 240 h. Overall, the results demonstrate that the maximum rate of metacyclogenesis of the resistant strains, AmpBRP2i and LSCM1-6, was rather similar to that of the LSCM1 parental strain but the differences observed at 144 and 168 h of culture were, however, considered statistically significant.

### *In vitro* PEM infection and intracellular amastigote multiplication

**Figure 4:**
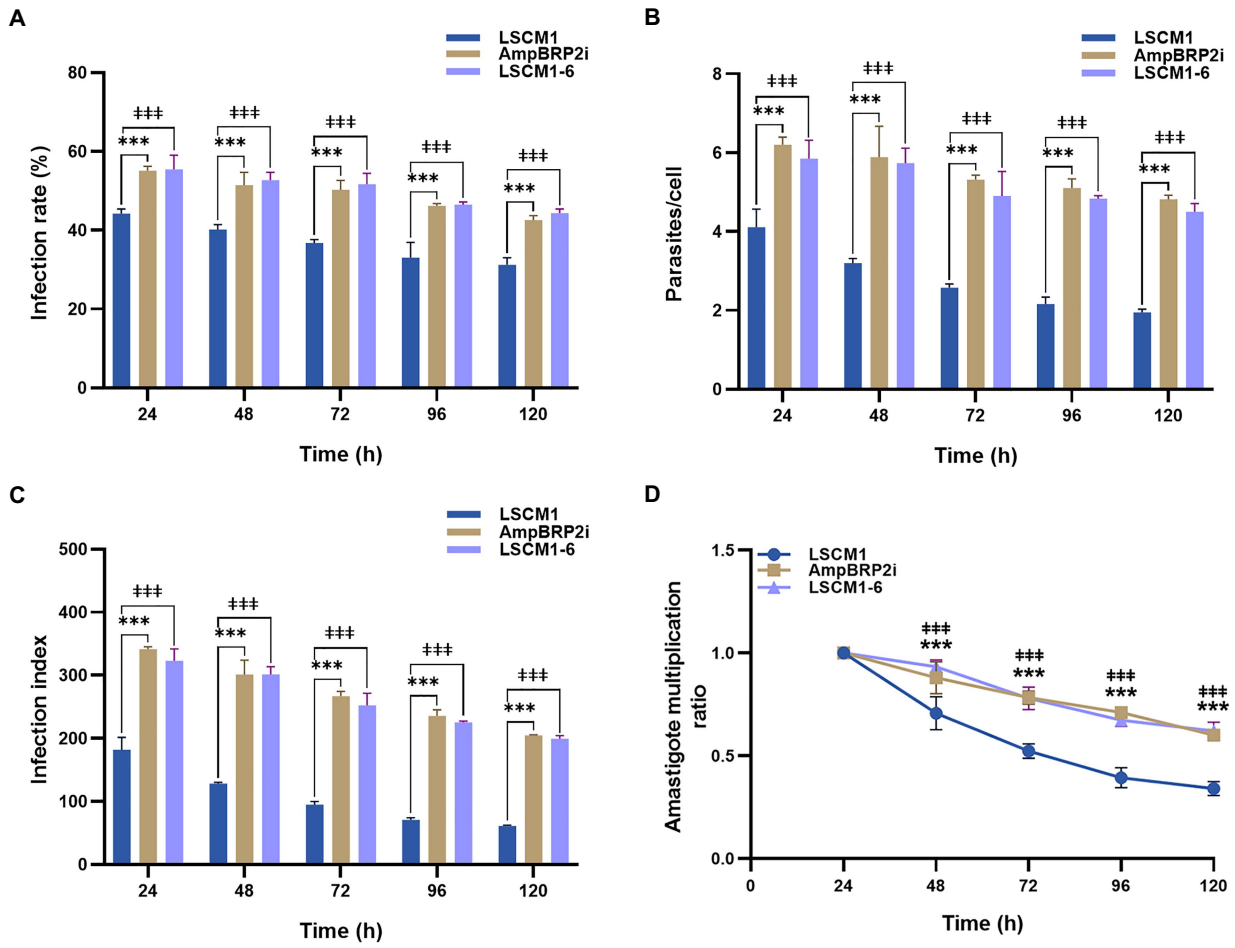
PEMs were infected using stationary phase promastigotes after 120 h of culture at a multiplicity of infection (MOI) = 1:10. **(A)** Percentage of infected cells measured from 24 to 120 h post-infection. **(B)** Average number of parasites per macrophage. **(C)** Infection index calculated by multiplying the percentage of infected macrophages by the average number of parasites per macrophage. **(D)** Amastigote multiplication ratio calculated from the average number of intracellular amastigotes at T_x_ divided by the average number of intracellular amastigotes at T_0_ (Chanmol et al., 2019). Results are expressed as the means±SD from three different experiments run in duplicate. Statistically significant differences between LSCM1 and AmpBRP2i are indicated as *** = *p* < 0.001; between LSCM1 and LSCM1-6 are indicated as **ǂǂǂ** = *p* < 0.001.

PEMs recovered 48 h after injection of Brewer thioglycolate were infected at a ratio of 1:10 using stationary phase promastigotes collected after 120 h of culture when the average number of metacyclic forms was similar for all the parasites. At all-time points of the infection, the infection rate, average number of intracellular parasites per cell, and infection index of the AmpBRP2i and LSCM1-6 strains were significantly higher than those of the LSCM1 strain (Figures 4A–C; Supplementary Table S3). The lesser uptake (1.6-fold) of the LSCM1 parasites alone cannot account for differences in infectivity that we observed. However, after 24 h post-infection, the infection index and the amastigote multiplication ratio of all strains gradually declined until 120 h of infection (Figures 4C,D) showing that none of the parasites replicated as amastigotes in PEMs. The failure to obtain a robust and long-lasting infection may result from the nature of the PEMs we used in our assay. Indeed, (i) mouse macrophages may not be as permissive for *L. martiniquensis* replication, and (ii) the use of thioglycolate elicits inflammatory macrophages with increased phagocytic and respiratory burst capacity. In conclusion, in our experimental *in vitro* system, the AmpB-resistant parasites were initially more infectious than the initial LSCM1 with more infected cells, more parasites per cell, and a persisting infection but as the susceptible parasites they fell to replicate.

### *In vivo* infectivity

**Figure 5:**
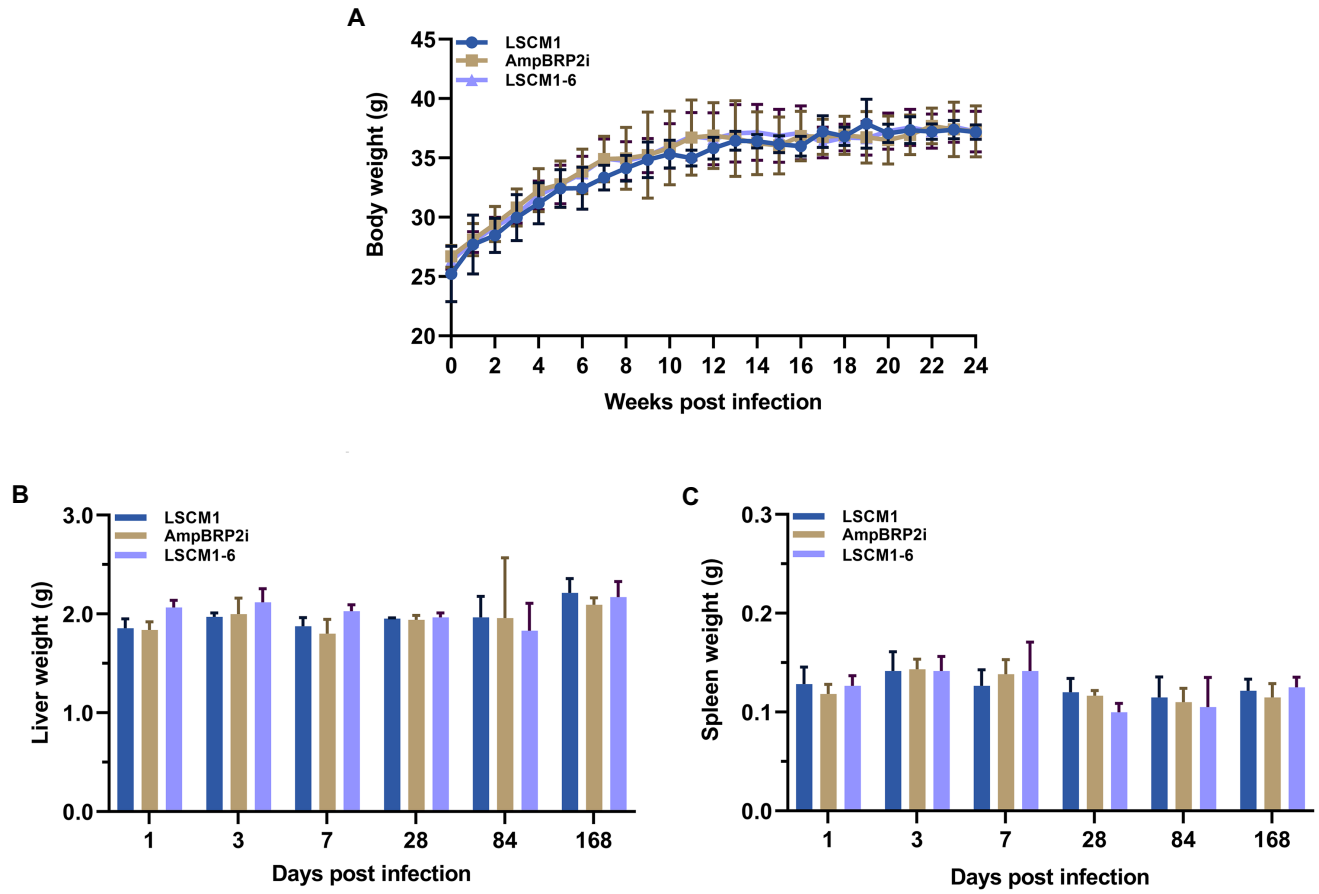
**(A)** Bodyweight of BALB/c mice infected with the *L. martiniquensis* LSCM1, AmpBRP2i, and LSCM1-6 stationary promastigotes. Animal weight was recorded every week. **(B,C)** Weight of liver and spleen collected from BALB/c mice infected with the *L. martiniquensis* LSCM1, AmpBRP2i, and LSCM1-6 stationary promastigotes.

BALB/c mice were inoculated with 2 × 10^7^ stationary phase culture promastigotes (120 h) of the LSCM1, AmpBRP2i, and LSCM1-6 strains, each composed of approximately 25% metacyclic promastigotes (Supplementary Table S2). Throughout the observation, no clinical signs of the disease including cachexia, fatigue, ascites, scabs or skin lesions, hepatomegaly, and splenomegaly, and no statistically significant differences in the weight of the body, liver, and spleen among the infected mice with the LSCM1, AmpBRP2i, and LSCM1-6 were observed (Figure 5). At all-time points, no parasites could be observed in any impression smear of any infected mice.

**Figure 6:**
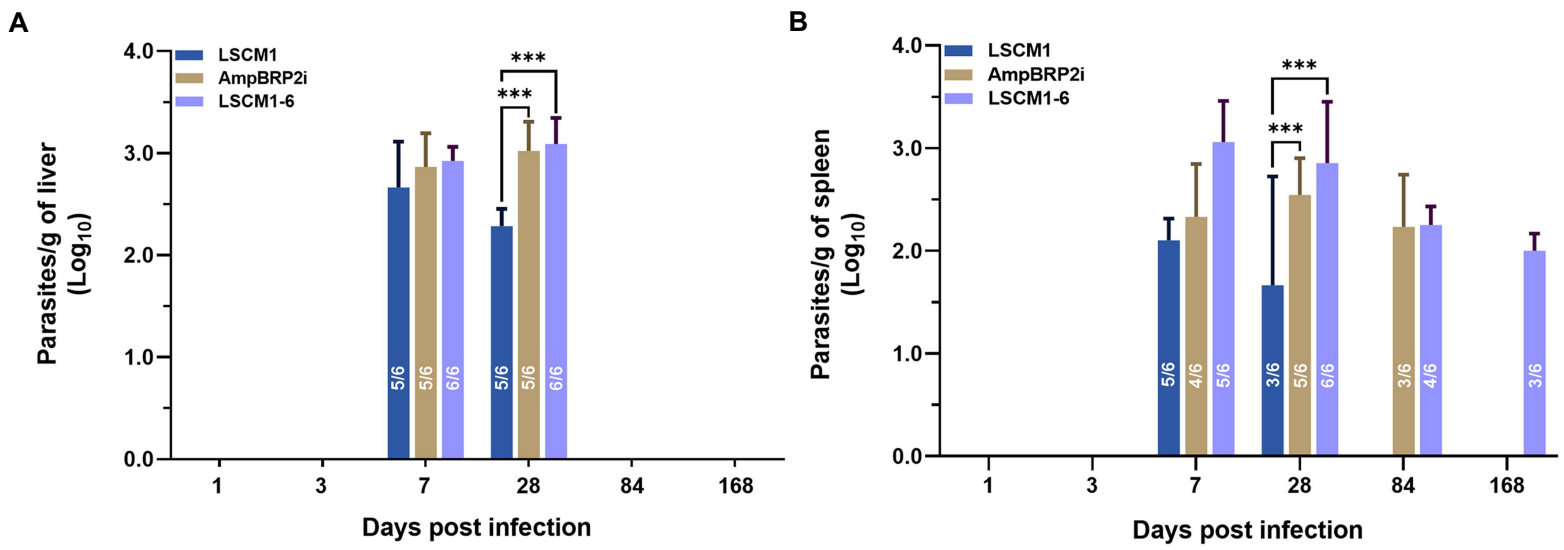
Parasite burdens in the liver and spleen samples of BALB/c mice infected with the *L. martiniquensis* LSCM1, AmpBRP2i, and LSCM1-6 stationary phase promastigotes at 1, 3, 7, 28, 84, and 168 dpi determined by limiting dilution assay. **(A)** Parasite load quantified from the liver of infected mice. **(B)** Parasite load quantified from the spleens of infected mice. Statistically significant differences among the three groups are indicated as follows: *** = *p* < 0.001. The number inside the columns is the number of positive mice per total mice sacrificed in each group.

Parasite burdens in the infected organs were quantified using a limiting dilution assay. At 1 and 3 dpi, no parasites were present in the culture of liver and spleen samples. After 7 dpi parasites were found in the cultures of the liver samples from five to six infected mice with a mean parasite burden of approximately 4.47 × 10^2^ parasites/gram of liver, 7.08 × 10^2^ parasites/gram of liver and 8.51 × 10^2^ parasites/gram of liver from mice infected with LSCM1, AmpBRP2i and LSCM1-6 parasites, respectively. At 28 dpi, the number of mice with parasites in the liver remained stable. The parasite load after infection with the LSCM1 started to decrease to 1.70 × 10^2^ parasites/gram of liver whilst in the livers of mice infected with the AmpB resistant parasites it slightly increased with 1.15 × 10^3^ and 1.44 × 10^3^ parasites/gram of organ for AmpBRP2i and LSCM1-6 parasites, respectively. Although the parasitic loads in the liver were low the difference between susceptible and resistant parasites is statistically significant. No parasites of any strains were isolated from the liver samples of the infected mice at 84 and 168 dpi (Figure 6A).

For the cultures of spleen samples, the LSCM1 parasites were recovered from infected mice sacrificed at 7 and 28 dpi with the parasitic load decreasing from 1.23 × 10^2^ (5 out of 6 mice) to 46.77 parasites/gram of organ in half of the animals After 84 and 168 dpi LSCM1 parasites were not anymore detected. AmpBRP2i parasites were found in the spleen of the mice sacrificed at 7 to 84 dpi with a parasite burden around 1.70 × 10^2^, 3.47 × 10^2^, and 1.70 × 10^2^parasites/gram of organ, respectively, in at least 3 to 5 mice. No AmpBRP2i parasites were observed in the samples at 168 dpi. For LSCM1-6, parasites were found in the cultures of the spleen samples collected at 7 to 168 dpi but the number of mice with parasites decreased over time with only half of them still carrying parasites in their spleen at the end of the experiment. Consequently, the parasite burden gradually decreased from 1.15 × 10^3^ to 1.23 × 10^2^ parasites/gram of organ (Figure 6B). No parasites were isolated from all bone marrow samples of the infected mice at all-time points.

Polymerase chain reactions on genomic DNA extracted from all liver spleen and bone marrow samples from the infected mice were performed. DNA of each parasite strain was detected in the same samples where parasites were quantified by the limiting dilution assay. No parasitic DNA could be detected in any of the bone marrow samples (Supplementary Figure S1).

The susceptibility to AmpB of the LSCM1, AmpBRP2i, and LSCM1-6 strains after the infection was tested. The IC_50_ value and resistance index at 28 dpi were not affected with 0.053 ± 0.01 and 1, 0.19 ± 0.02 and 3.6, and 0.154 ± 0.08 and 2.9, respectively, indicating that the resistant phenotype is stable (Table 2).

Even though the mice model may not be the more suitable system for experimental infection with *L. martiniquensis*, our results suggest that AmpB-resistant parasites may survive longer in the animals.

## Discussion

Drug resistance in *Leishmania* parasites is frequently associated with molecular and fitness changes. AmpB resistance in *L. martiniquensis* and its consequences on the parasite phenotype is poorly documented especially in clinical samples due to the (i) recent identification of this new species, (ii) the small number of patients with a positive diagnosis of leishmaniasis and AmpB treatment failure, and (iii) the even smaller number of patients from whom parasites could be isolated before and after the relapse. We have reported three cases of leishmaniasis caused by *L. martiniquensis* in northern Thailand that showed relapse of the disease after the first treatment with AmpB (Pothirat et al., 2014; Chiewchanvit et al., 2015). We successfully isolated the parasites from a patient before treatment (LSCM1) and after relapse (LSCM1-6) and could generate an *in vitro*-induced AmpB-resistant clone (AmpBRP2) that was subsequently inoculated to mouse (AmpBRP2i). Here, we used phenotypic analyses to correlate AmpB resistance with fitness changes that could be relevant for parasite transmission or survival.

The inoculation in the mouse of the AmpBRP2 clone that was maintained for 40 *in vitro* passages led to the recovery of parasites (AmpBRP2i) with an 8-fold decrease in the IC_50_ compared to the initial clone. Furthermore, the IC_50_ value and resistance index were unchanged even after a second infection in mice revealing the stability of the phenotype. It suggests that the AmpBRP2i strain was a good representative for *in vitro*-generated AmpB-resistant strains and could be used the same parameters to analyze the influence of drug resistance on the fitness of the resistant strain in order to compare with the wild-type LSCM1 and the relapse LSCM1-6 strains. The difference in the level of resistance between the AmpBRP2 and AmpBRP2i is not associated with the absence of drug pressure during the passage in the animal since AmpBRP2 was maintained *in vitro* in a drug-free medium for 40 passages before the injection to the mouse. *Leishmania* genomic plasticity may account for this result as drug resistance (reviewed by Rastrojo et al., 2018; Santi and Murta, 2022) and long-term culture (Dumetz et al., 2017; Prieto Barja et al., 2017) has been associated with chromosome and gene copy number variations or down-regulation of genes. In AmpB-resistant *L. donovani* parasites generated *in vitro,* chromosome 29 for which many transcripts were overexpressed is co-amplified with chromosomes 5 and 26 (Rastrojo et al., 2018) previously associated with fast growth in culture (Dumetz et al., 2017; Prieto Barja et al., 2017). Conversely, inoculation to animals of trisomic parasites notably for chromosomes 5 and 26 resulted in a shift to disomic karyotype (Prieto Barja et al., 2017). Comparative genomic analysis of AmpBRP2 and AmpBRP2i would be needed to confirm this hypothesis. Such chromosome amplification may also explain the differences in growth that we observed between AmpB-resistant (AmpBRP2i and LSCM1-6) and the parental strain (LSCM1).

The naturally resistant strain (LSCM1-6) and the *in vitro*-induced AmpB-resistant clone after passage in the mouse (AmpBR2i) display the same trend for all the parameters we have tested including IC_50_ suggesting that the molecular mechanisms underlying the resistance to the drug are if not similar at least converge to the same parasite phenotype. Pountain et al. (2019) have shown that AmpB *L. mexicana-*resistant lines could harbor mutation(s) in one or more genes from the sterol pathway. At least three genes in which mutations resulted in AmpB resistance in *L. mexicana* have been identified, i.e., genes involving (1) resistance-associated mutation of the C5DS, (2) specific sterol changes resulting from decreased expression of the C24SMT due to structural variation events at the genome level, and (3) loss of the miltefosine transporter (Pountain et al., 2019). Recently, Alpizar-Sosa et al. (2022) have reported the selection and characterization of fourteen independent lines of *L. mexicana* and one of *L. infantum* resistant to AmpB or its analog nystatin and demonstrated loss of heterozygosity derived from mutations in the C24SMT gene locus and changes in the C5DS gene. Single-cell DNA/RNAseq of our naturally resistant parasites (LSCM1-6) would help to characterize the molecular basis of AmpB resistance in the context of a treatment failure.

Drug-resistant parasites tend to be less infective, less virulent or display a decreased transmission potential (Hendrickx et al., 2015) or at best they present mixed fitness gain in mammal and sand fly hosts compared to WT strain (Hendrickx et al., 2020; Van Bockstal et al., 2020). In our study, in contrast, the fitness of the *L. martiniquensis* AmpB-resistant parasites (LSCM1-6 and AmpBPR2i) increased compared to the wild-type LSCM1. The AmpBRP2i and the LSCM1-6 grew at higher concentrations and produced more metacyclic forms than the LSCM1. The fitness of *Leishmania* parasites relates to their ability to successfully survive, reproduce/replicate, infect, and - be transmitted from the host to the vector and reciprocally (Natera et al., 2007). Metacyclogenesis is regarded as a contributor to the fitness of the parasite but the ability of metacyclic promastigotes themselves is an important factor supporting the successful infectivity of the parasites. However, immunopathology depends on the ability of metacyclic forms to undergo differentiation in replicating amastigotes and the genetic background/immune status of the host. BALB/c mouse model is a suitable model for the asymptomatic form of human leishmaniasis caused by *L. martiniquensis* whilst hamsters are symptomatic (Intakhan et al., 2020). In our study, when injected into BALB/c mice the AmpB-resistant parasites promoted a persisting infection, although at a low level, compared to the WT strain. This silent infection could greatly impact the level of transmission of *L. martiniquensis* AmpB-resistant parasites in the field provided that AmpB resistance does not affect infectivity in vectors.

In conclusion, our results provide indications that AmpB treatment of leishmaniasis caused by *L. martiniquensis* has the potential to generate parasites with increased fitness, not only in response to treatment (hence resistance) but also in terms of infectivity and transmission. In Thailand, at least 24.9% of HIV/*Leishmania* coinfected patients are asymptomatic and *L. martiniquensis* is one of the predominant species detected (Manomat et al., 2017). Since not only symptomatic leishmaniasis patients but also asymptomatic HIV/leishmaniasis patients play an important role as “reservoirs” in *Leishmania* transmission (Molina et al., 2020; Ibarra-Meneses et al., 2022), our results highlight the need to search and monitor for AmpB-resistant *L. martiniquensis* in both patients with symptomatic and asymptomatic leishmaniasis, mainly in HIV/*Leishmania* coinfected patients.

## Data availability statement

The original contributions presented in the study are included in the article/Supplementary material, further inquiries can be directed to the corresponding author.

## Ethics statement

The studies involving human participants were reviewed and approved by The ethics committee of the Faculty of Medicine, Chulalongkorn University (COA No. 467/2021). Written informed consent for participation was not required for this study in accordance with the national legislation and the institutional requirements. The animal study was reviewed and approved by The Ethics Committee on Animal Use of the Laboratory Animal Center, Chiang Mai University, Chiang Mai, Thailand (COA No. 2562/MC-0009).

## Author contributions

NJ and CM: conceptualization, methodology, formal analysis, and writing-original draft preparation. CM, AK, AT, and NJ: investigation. CM and NJ: visualization. NJ, SR, CU, PP, and GS: writing-review and editing. NJ, PT, PrS, PaS, and GS: funding acquisition. All authors contributed to the article and approved the submitted version.

## Funding

This work was supported by Ratchadapiseksompotch Fund Chulalongkorn University [grant number RCU_H_64_013_30] received by NJ; the Thailand Graduate Institute of Science and Technology (TGIST) and NSTDA [grant number TG-22-10-60-001D] received by NJ for CM; the Faculty of Medicine, Chiang Mai University [grant number 128–2562] received by PT; the Program Management Unit for Human Resources & Institutional Development, Research and Innovation - Chulalongkorn University [grant number B16F630071] received by PaS; the Thailand Science Research and Innovation (TSRI) Fund [grant number CU_FRB640001_01_30_1] received by PaS and NJ; the French Ministry of Europe and Foreign Affairs (MEAE), the French Ministry of Higher Education, Research and Innovation (MESRI) and the Ministry of Higher Education, Science, Research and Innovation of Thailand (MHESI) - Franco-Thai Mobility Program/PHC SIAM 2021–2022 [grant number PHC SIAM 2021–2022] received by NJ and GS.

## Conflict of interest

The authors declare that the research was conducted in the absence of any commercial or financial relationships that could be construed as a potential conflict of interest.

## Publisher’s note

All claims expressed in this article are solely those of the authors and do not necessarily represent those of their affiliated organizations, or those of the publisher, the editors and the reviewers. Any product that may be evaluated in this article, or claim that may be made by its manufacturer, is not guaranteed or endorsed by the publisher.
