# Supplementary material for: Amphotericin B resistance correlates with increased fitness in vitro and in vivo in Leishmania (Mundinia) martiniquensis
Source: Front Microbiol. 2023 Apr 6;14:1156061. doi: 10.3389/fmicb.2023.1156061 (PMC10116047; doi:10.3389/fmicb.2023.1156061)
Supplement: Supplementary file 1 [file Data_Sheet_1.docx]

Supplementary Material

**Amphotericin B resistance correlates with increased fitness *in vitro* and *in vivo* in *Leishmania* (*Mundinia*) *martiniquensis***

**Chonlada Mano^1^, Aphisek Kongkaew^2^, Pongsri Tippawangkosol^3^, Pradya Somboon^3^, Sittiruk Roytrakul^4^, Pascale Pescher^5^, Gerald F. Späth^5^, Chairat Uthaipibull^4,6^, Adisak Tantiworawit^7^, Padet Siriyasatien^8^, Narissara Jariyapan^8^***

*** Correspondence:** Narissara Jariyapan: [narissara.j@chula.ac.th](mailto:narissara.j@chula.ac.th)


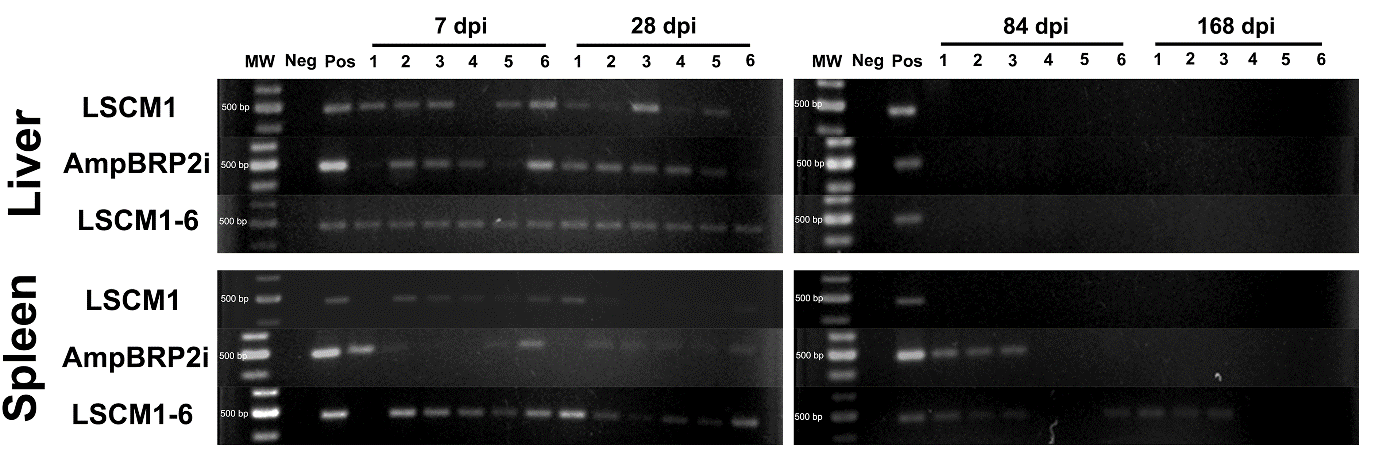


**Supplementary Figure 1.** PCR amplification of *L. martiniquensis* DNA in liver and spleen samples of infected BALB/c mice using 70IRD/70IRM primers. Liver and spleen samples were collected at 7, 28, 84, and 168 dpi. Lanes: MW, 100 bp DNA ladder; Neg, negative control (no DNA); Pos, positive control (*L. martiniquensis* DNA); 1- 6, samples from mouse number 1-6 at 7, 28, 84, and 168 dpi.

**Supplementary Table 1.** Growth of the LSCM1, AmpBRP2i, and LSCM1-6 promastigotes. Results are expressed as mean±SD based on three independent replicates.

| Time in culture (h) | Number of promastigotes (×10^7^ cell/mL) | | |
| --- | --- | --- | --- |
|  | LSCM1 | AmpBRP2i | LSCM1-6 |
| 24 | 0.29±0.58 | 0.24±0.68 | 0.37±0.92 |
| 48 | 0.75±0.74 | 1.00±0.79 | 1.14±0.68 |
| 72 | 2.87±1.34 | 3.25±2.58 | 3.35±4.31 |
| 96 | 4.55±3.04 | 4.13±5.21 | 5.54±5.21 |
| 120 | 4.56±3.34 | 6.91±0.18 | 6.35±5.32 |
| 144 | 4.23±1.06 | 6.82±2.01 | 5.84±4.62 |
| 168 | 2.35±0.67 | 5.35±2.89 | 4.32±13.78 |
| 192 | 1.59±1.92 | 4.09±1.53 | 3.83±4.00 |
| 216 | 0.69±1.96 | 2.79±4.40 | 1.69±6.30 |
| 240 | 0.32±0.32 | 1.97±0.94 | 1.36±2.51 |

**Supplementary Table 2.** Percentage of metacyclic promastigotes for the LSCM1, AmpBRP2i, and LSCM1-6 strains. Results are expressed as mean±SD based on three independent replicates.

| Time in culture (h) | % Metacyclic promastigotes | | |
| --- | --- | --- | --- |
|  | LSCM1 | AmpBRP2i | LSCM1-6 |
| 72 | 3.61±0.25 | 5.39±1.13 | 4.33±0.87 |
| 96 | 16.72±0.84 | 17.22±1.23 | 17.06±0.86 |
| 120 | 25.39±1.25 | 26.94±0.86 | 25.50±1.33 |
| 144 | 25.28±1.35 | 27.61±1.75 | 28.06±1.00 |
| 168 | 23.06±0.92 | 26.00±0.50 | 26.00±0.44 |
| 192 | 17.67±1.04 | 18.61±1.13 | 18.17±0.44 |
| 216 | 14.17±1.09 | 15.44±0.25 | 15.72±0.82 |
| 240 | 9.72±1.29 | 12.06±1.11 | 12.22±1.84 |

**Supplementary Table 3.** The infection rate, average number of intracellular parasites per macrophage, infection index, and intracellular amastigote multiplication ratio of the LSCM1, AmpBRP2i, or LSCM1-6 strains. Results are expressed as mean±SD based on three independent replicates.

| Time post infection (h) | Infection rate (%) | Average number of intracellular parasites/macrophage | Infection index | Amastigote multiplication ratio |
| --- | --- | --- | --- | --- |
| LSCM1 | | | | |
| 24 | 44.25±1.15 | 4.11±0.46 | 181.75±19.72 | 1.00±0.00 |
| 48 | 40.17±1.28 | 3.19±0.12 | 128.08±2.02 | 0.71±0.08 |
| 72 | 36.75±0.90 | 2.58±0.09 | 94.67±5.06 | 0.52±0.03 |
| 96 | 33.00±3.93 | 2.16±0.18 | 70.83±2.93 | 0.39±0.05 |
| 120 | 31.25±1.75 | 1.95±0.08 | 61.00±1.39 | 0.34±0.03 |
| AmpBRP2i | | | | |
| 24 | 55.08±1.13 | 6.20±0.20 | 341.58±3.79 | 1.00±0.00 |
| 48 | 51.42±3.30 | 5.89±0.78 | 301.08±22.88 | 0.88±0.08 |
| 72 | 50.25±2.41 | 5.32±0.11 | 266.92±7.46 | 0.78±0.03 |
| 96 | 46.17±0.63 | 5.10±0.24 | 235.50±9.82 | 0.69±0.02 |
| 120 | 42.58±1.13 | 4.81±0.11 | 204.75±0.90 | 0.60±0.01 |
| LSCM1-6 | | | | |
| 24 | 55.42±3.64 | 5.85±0.47 | 323.17±18.51 | 1.00±0.00 |
| 48 | 52.67±2.04 | 5.73±0.39 | 301.50±12.05 | 0.93±0.03 |
| 72 | 51.67±2.74 | 4.90±0.62 | 252.17±19.38 | 0.78±0.05 |
| 96 | 46.50±0.66 | 4.84±0.07 | 225.08±2.31 | 0.70±0.03 |
| 120 | 44.33±1.01 | 4.50±0.21 | 199.33±5.13 | 0.62±0.04 |
